# The energy consumption structure and African EMDEs' sustainable development

**DOI:** 10.1016/j.heliyon.2020.e03822

**Published:** 2020-04-26

**Authors:** Hoang Phong Le, Dang Thi Bach Van

**Affiliations:** a School of Public Finance, University of Economics Ho Chi Minh City, 59C Nguyen Dinh Chieu, District 3, Ho Chi Minh City, Viet Nam; b Department of Finance and Accounting Management, Ho Chi Minh City University of Law, 02 Nguyen Tat Thanh Street, District 4, Ho Chi Minh City, Viet Nam

**Keywords:** Energy, Economics, Economic development, Economic growth, Macroeconomics, Econometrics, Energy economics, African EMDEs, Renewable power and conventional fuels consumption, Sustainable development

## Abstract

This research evaluates the importance of renewable power and conventional fuels consumption in the economic growth of 20 African EMDEs towards sustainable development. Due to the evidence of slope heterogeneity alongside cross-sectional dependence, the author applies second-generational econometric techniques for heterogeneous panel data. After detecting the long-term relationship among all variables using Westerlund panel-data cointegration test, the long-run estimates are computed by AMG, MG and CCEMG estimators, which indicates that nonrenewable and renewable energy usage fosters African EMDEs' economic growth. Besides, capital, government expenditure, and trade openness also encourage economic growth. Moreover, the causality analysis (using Dumitrescu and Hurlin test) supports the feedback effects among the selected variables and economic growth. The findings provide critical implications for sustainable energy policies that contribute to the sustainable development of African EMDEs.

## 1 Introduction

Almost developed and developing countries need energy for their industrialization and socio-economic development, which results in the upsurge of energy consumption together with lower and lower environmental quality (Shahbaz et al., 2017; Phong et al., 2018; Phong, 2019). Conventional energy use from fossil fuel sources is deemed an important factor causing greenhouse emissions and threatening sustainable development (Kaygusuz, 2007; Gozgor et al., 2018). In the circumstance of escalating environmental degradation, developed and developing countries are heading towards “green” programs. It is obvious that one of the main focuses of these programs is the exploitation of renewable and clean energy sources (Jacobs, 2012; Hodbod and Tomei, 2013), which can reduce climate change and contribute to sustainable development (Bhattacharya et al., 2017; Goh and Ang, 2018). Moreover, the usage of renewable energy was also among the 17 sustainable development objectives proposed by the United Nations in 2015 (Singh et al., 2019). The growth of renewable energy has created a great opportunity for the transition of energy structure in recent years, which is attributed to technology advancement as well as cost reduction in renewable energy. According to IEA, wind and solar PV power are estimated to occupy more than 50% of the new energy supply until 2040 in the Stated Policies Scenario and they will contribute nearly 100% of the growth in the Sustainable Development Scenario (IEA, 2020). Consequently, many countries in the world have paid attention to the role of renewable energy usage in sustainable development as well as the adjustment of energy consumption structure. Likewise, African countries, especially the Emerging Market and Developing Economies (EMDEs), are actively transitioning their energy structures in order to pursue “green” economies when the energy demand for economic and population growth is putting more and more pressure and the threat of energy insecurity combined with loose regulations for protecting the environment is persistent (Saidi and Mbarek, 2017; Slesman et al., 2019). This motivates researches on the relationship between the energy consumption structure and sustainable development.

Although the linkage between energy usage and economic growth has been documented by numerous studies in the past four decades, it remains the center of controversy regarding energy policies (Apergis and Payne, 2009a,b,c; Payne, 2010; Ozturk, 2010). And identifying the aforesaid linkage is considered a key to energy policies (Ozturk, 2010). Thus, the focus of empirical studies on the relationship between energy consumption and economic growth should answer these crucial research questions: How does energy usage have a long-run equilibrium relationship with economic growth? What are the causality mechanisms between energy utilization and economic growth? This is very essential for policy makers and other stakeholders to design and implement effective energy policies towards the sustainable development goal.

Based on the aforesaid explanation, the objective of this study is examining the long-run relationship and the causality between renewable and nonrenewable energy consumption and the economic growth in African Emerging Market and Developing Economies (EMDEs) from 1990 to 2014. Based on the research objective, the hypotheses of this study are: (i) Renewable power consumption and nonrenewable energy usage positively affect economic growth; (ii) The feedback hypothesis (bidirectional causation between renewable and nonrenewable power usage and economic growth) is supported. In order to conduct this empirical study, we apply “second-generation” econometric techniques to solve the heterogeneous panel problem for the model based on the extended version of the Cobb-Douglas production function.

This study contributes to the existing literature in several ways. First, EMDEs in general and African EMDEs in particular are rapidly integrated with the global economy; nevertheless, they face the challenges of power security alongside weak standards of environmental-protection (Saidi and Mbarek, 2017; Slesman et al., 2019). From the policy viewpoint, the identifying the relationship between energy and growth is deemed a crucial factor in making energy policies (Ozturk, 2010). This study focuses on the examination of the long-run impacts of both renewable and nonrenewable energy sources on economic growth as well as the causal relationship among these variables in African EMDEs. Hence, this article is meaningful to the policy-makers and stakeholders in identifying strategies for building sustainable energy structure so as to contribute to the sustainable development goal of African EMDEs. Second, despite a large body of empirical documents about the linkage between energy consumption and economic growth, to the best of our knowledge, it seems that there is a research gap for the case of African EMDEs. Third, this article extends the production function, which considers the effects of power consumption (in terms of renewable and nonrenewable energy usage) on economic growth alongside other explanatory variables including capital, trade openness and government expenditure. This avoids omitting important variables and generates more robust results. Besides, this paper applies a new approach known as second-generational econometric techniques that effectively deals with cross-sectional dependence (CSD) and slope heterogeneity, thus providing more robust estimates in comparison with first-generation econometric techniques that ignore CSD and slope heterogeneity.

The main findings of this study include: (1) Nonrenewable energy usage per capita and renewable energy consumption per capita stimulate GDP per capita. (2) There exist two-way relationship between nonrenewable energy usage and GDP per capita as well as between renewable energy usage and GDP per capita, thus demonstrating the presence of the feedback hypothesis between the two components of energy consumption (nonrenewable fuels and renewable power) and economic growth. (3) This study witnesses that gross fixed capital formation per capita, the government expenditure per capita and the trade openness per capita positively affect GDP per capita. Besides, the feedback effects among those variables and economic growth. (4) The analysis results of AMG, MG and CCEMG estimators are consistent and very similar in terms of the signs and magnitudes of the coefficients, which signifies the robustness of estimation.

The rest of this article is as follows: Section 2 provides literature review, Section 3 presents the empirical model, data, and methodology, Section 4 shows and explains the findings, and finally section lists essential conclusion as well as recommendations for policy-makers.

## 2 Literature review

Since the study of Kraft and Kraft (1978) on the connection between power usage and economic growth, this topic has been widely researched in the past decades and remained a hot and controversial topic in energy economics discipline because the evidences are uncertain (or unclear) when discovering the energy-growth relationship in different countries or groups of countries due to different time periods, different variables and the weaknesses of econometric methods (Ozturk, 2010; Payne, 2010; Menegaki, 2014; Omri, 2014; Ahmad at al., 2020). Generally, the available literature provides four hypotheses to explain the connection between energy use and economic growth: (1) *The growth hypothesis* mentions the unidirectional effect of energy usage on economic growth that higher energy utilization facilitates more economic activities. Thus, energy conservation policies are deemed having negative impacts on economic growth. (2) *The conservation hypothesis* assumes one-way causation from economic growth to energy consumption. In other words, more economic activities boost energy usage. Hence, energy conservation policies do not affect economic growth. (3) *The feedback hypothesis supposes* bidirectional causality between energy usage and economic growth. Accordingly, energy conservation policies can affect economic growth efficiency, and economic growth can also influence energy consumption. (4) *The neutrality hypothesis* argues that energy consumption and economic growth have no impact on each other. Consequently, energy conservation policies aiming to reduce power consumption have no influence on economic growth.

Based on the literature review, it can be observed that there are 3 branches of research regarding the association between energy consumption and economic growth. The first one is about studying the relationship between all energy usage and economic growth. The second one is about examining the linkage between renewable energy consumption and economic growth. And the final one is about studying the connection between the consumption of 2 energy types (non-renewable and renewable) and economic growth. Table 1 below presents some recent researches from the 3 aforementioned branches.

**Table 1:** Summary of recent researches.

Author(s)	Countries	Period	Methodology	Results
The literature on the linkage between economic growth and energy consumption				
Ozturk et al. (2010)	51 countries	1971–2005	FMOLS; DOLS; Granger causality	Feedback and Conservation
Pao and Tsai (2011)	BRIC	1980–2007	VECM	Feedback
Yildirim and Aslan (2012)	17 highly developed OECD countries	1964–2010	Toda-Yamamoto procedure; the bootstrap-corrected causality	Growth and Feedback
Shahbaz et al. (2013)	China	1971–2011	ARDL; VECM	Growth
Kumar et al. (2014)	Albania, Bulgaria, Hungary and Romania	1971–2011	ARDL; Toda and Yamamoto causality	Conservation
Nasreen and Anwar (2014)	15 Asian countries	1980–2011	DOLS; FMOLS; VECM	Feedback
Yıldırım et al. (2014)	the Next 11 countries	1971–2010	VAR; the bootstrapped AR metric causality	Growth and Neutrality
Azam et al. (2015)	ASEAN-5 countries	1980–2012	VAR; Granger causality	Growth and Neutrality
Adewuyi and Awodumi (2017)	106 countries	1971–2011	VAR; Granger causality	Feedback

The literature on the linkage between economic growth and renewable energy consumption				
Apergis and Payne (2010)	20 OECD	1985–2005	FMOLS; VECM	Feedback
Menegaki (2011)	27 European countries	1997–2007	One-way random effect model; Panel Causality	neutrality
Salim and Rafiq (2012)	18 emerging countries	1994–2003	FMOLS; DOLS; Granger causality	Conservation
Bildirici (2013)	10 developing and emerging countries	1980–2009	ARDL; VECM	Growth and Feedback
Zeb et al. (2014)	SAARC	1975–2010	FMOLS; VECM	Growth
Alper and Oguz (2016)	EU member countries	1990–2009	ARDL; Hatemi-J causality	Growth, Conversation and Neutrality
Aslan (2016)	United States	1961–2011	ARDL	Growth
Cetin (2016)	E-7	1992–2012	FMOLS; DOLS; Granger causality	Neutrality
Menegaki and Ozturk (2016)	MENA	1997–2009	FE; Granger causality	Growth
Ohlan (2016)	India	1971–2012	ARDL; VECM	Growth and Feedback
Tugcu and Tiwari (2016)	BRICS	1992–2012	Panel bootstrap Granger causality	Neutrality
Carmona et al. (2017)	USA	1973–2015	Toda-Yamamoto causality	Neutrality
Destek and Aslan (2017)	Emerging economies	1980–2012	Bootstrap panel causality	Feedback, Neutrality, Conservation, and Growth
Furuoka (2017)	Baltic region	1992–2011	Granger causality; Dumitrescu-Hurlin panel causality	Conservation
Shakouri and Khoshnevis Yazdi (2017)	South Africa	1971–2015	ARDL; Granger causality	Feedback

Brief literature on both renewable and nonrenewable energy consumption-economic growth nexus				
Payne (2009)	US	1949–2007	Toda-Yamamoto causality	Neutrality
Apergis and Payne (2011)	80 countries	1990–2007	FMOLS; DOLS; VECM	Feedback
Apergis and Payne (2012)	Central America	1990–2007	VECM	Growth and Feedback
Tugcu et al. (2012)	G7	1980–2009	ARDL; Hatemi-J causality	Conservation, Growth, Feedback, and Neutrality
Al-Mulali et al. (2014)	18 Latin American countries	1980–2010	DOLS; VECM	Feedback
Jebli and Youssef (2015)	69 countries	1998–2010	OLS; FMOLS; DOLS; VECM	Neutrality
Kahia et al. (2017)	11 NOICs countries	1980–2012	FMOLS; VECM	Feedback
Ben Mbarek et al. (2018)	Tunisia	1990–2015	Granger causality; VECM	Conservation and Growth
Ahmed et al. (2019)	Myanmar	1990–2016	ARDL; DOLS; FMOLS; VECM	Growth
Bekun et al. (2019)	EU-16 countries	1996–2014	Dumitrescu and Hurlin Panel causality	Feedback
Le and Sarkodie, 2020	45 Emerging Market and Developing Economies	1990–2014	AMG, CCEMG and MG estimators; Dumitrescu-Hurlin panel causality	Feedback
Le and Bao (2020)	16 Latin America and Caribbean countries	1990–2014	AMG, CCEMG and MG estimators; Dumitrescu-Hurlin panel causality	Feedback
Rahman and Velayutham (2020)	5 South Asian countries	1990–2014	FMOLS; DOLS; Dumitrescu-Hurlin panel causality	Conservation

Notes: AMG: Augmented mean group; ARDL: autoregressive distributed lag; CCEMG: Common correlated effects mean group; CUP-BC: Continuously updated bias-corrected; CUP-FM: Continuously updated fully modified ordinary least square; DOLS: Dynamic ordinary least squares; ECM: Error correction model; FMOLS: Fully modified OLS; GMM: generalized methods of moments; MG: Mean group; OLS: Ordinary least squares; VAR: Vector autoregressive model; VECM: Vector error correction model.

As the research objective relates to the inspection and comparison of the impacts of renewable energy usage and nonrenewable energy consumption on economic growth as well as the detection of the causation between the energy variables and economic growth, we will present a thorough review of some notable studies in this research branch.

Payne (2009) examined the effects of renewable and nonrenewable power on the economic growth of the United States from 1949 to 2006 using Toda-Yamamoto causality method and supported the neutrality hypothesis (i.e. no causality between power consumption and economic growth). Apergis and Payne (2011) examined the relationship between the two types of energy (renewable and nonrenewable) usage and economic growth in 80 countries from 1990 to 2007. Results from the FMOLS model indicated that besides capital and labor, both renewable and nonrenewable energy positively influenced economic growth. The feedback hypothesis is confirmed by using VECM granger causality to test the causality between renewable and non-renewable energy usage and economic growth. Following this topic, Apergis and Payne (2012) inspected the link between renewable and nonrenewable electricity consumption and economic growth by employing VECM model for the panel data of Central American countries in the period 1990–2007. The findings showed that all variables except renewable electricity consumption stimulated economic growth. The causality test concerning the relationship between renewable electricity consumption and economic growth acknowledged the feedback hypothesis in the long-run but supported the growth hypothesis in the short-run. Regarding the relationship between nonrenewable electricity consumption and economic growth, the feedback hypothesis is validated in both the short-run and long-run.

Tugcu et al. (2012) assessed the connection between renewable energy and nonrenewable energy and economic growth in G7 countries from 1980–2009 by ARDL approach. The findings indicated that renewable or nonrenewable energy consumption promotes economic growth in the long-run. Besides, regarding the relationship between non-renewable energy consumption and economic growth, by using Hatemi-J causality analysis, the authors supported the growth hypothesis the in Japan and the neutrality hypothesis in the remaining countries. Meanwhile, concerning the relationship between renewable energy consumption and economic growth, the authors supported the feedback hypothesis in England and Japan, the conservation hypothesis in Germany and the neutrality hypothesis in France, Canada, Italy and the USA.

Al-mulali (2014) examined the impacts of renewable and nonrenewable electricity utilization on economic growth in 18 Latin American countries between 1980 and 2010. Results from DOLS model proved that renewable and nonrenewable electricity utilization, along with capital formation, labor, and trade, boosted economic growth in the long-run. Furthermore, VECM Granger causality tests indicated bidirectional causation among the variables.

Jebli and Youssef (2015) inspected the association between power consumption (renewable and nonrenewable) and economic growth in 69 countries in the period 1998–2010. The long-term-impact analyses using OLS, FMOLS and DOLS reported the positive effects of power consumption (renewable and nonrenewable), labor, export and import on economic growth. In addition, employing VECM Granger causality test, the authors concluded the neutrality hypothesis in the relationship between renewable energy usage and economic growth as well as the connection between nonrenewable energy usage and economic growth.

Kahia et al. (2017) investigated how renewable and nonrenewable energy utilization, along with other factors including capital and labor, affected economic growth in MENA Net Oil Importing Countries from 1980 to 2012 by using FMOLS and panel VECM granger causality. They found that both renewable and nonrenewable energy consumption encouraged economic growth. Moreover, the findings acknowledged the feedback hypothesis between each type of energy consumption and economic growth in the sample.

Ben Mbarek et al. (2018) inspected the connection between CO2 emissions, renewable and nonrenewable energy use, and economic growth in Tunisia by applying Granger causality test and VECM model for the time-series data in the period 1990–2015. The findings validated the growth hypothesis in the relationship between renewable energy use and economic growth as well as the conservation hypothesis in the association between nonrenewable energy use and economic growth. Also, this study supported renewable energy policy towards the sustainability and green economy in Tunisia.

Ahmed et al. (2019) studied the dynamic relationship between CO2 intensity, renewable and non-renewable energy usage, and economic growth in Myanmar from 1990 to 2016. The ARDL approach is used for analyzing the neo-classical growth model, while DOLS and FMOLS are employed for checking the results. The findings indicated that only renewable energy stimulated economic growth while the nonrenewable one produced negative impact due to inefficient technology. The causality analysis applying VECM granger causality indicated that growth hypothesis occurs in the relationship between each kind of energy usage and economic growth in Myanmar. Their findings emphasized that the generation and consumption of renewable power could contribute to the sustainable development in Myanmar.

Bekun et al. (2019) inspected the linkage between natural resources rent, renewable energy consumption, nonrenewable energy consumption, CO_2_ emissions and economic growth in EU-16 countries from 1996 to 2014. Dumitrescu and Hurlin Panel causality analysis indicated that a feedback mechanism among the consumption of renewable and nonrenewable power, and economic growth. Le and Sarkodie, 2020 documented the positive effects of nonrenewable and renewable power usage, capital, CO_2_ emissions, trade openness, and government expenditure on economic growth in 45 Emerging Market and Developing Economies during 1990–2014 by utilizing AMG, CCEMG and MG estimators. Furthermore, using Dumitrescu-Hurlin panel causality, their findings also supported the feedback hypothesis between nonrenewable and renewable energy usage and economic growth.

Le and Bao (2020) evaluated the connection between renewable and nonrenewable energy consumption and economic growth in 16 Latin American and Caribbean countries using AMG, CCEMG and MG estimators as well as Dumitrescu-Hurlin panel causality analysis on the panel data from 1990 to 2014. The findings indicated that both renewable and nonrenewable energy consumption promoted economic growth in the long-run. The feedback hypothesis was confirmed for each type of energy usage and economic growth.

Rahman and Velayutham (2020) applied FMOLS, DOLS and Dumitrescu-Hurlin panel causality in order to assess the long-run relationship as well as the causation between renewable and nonrenewable energy utilization and economic growth in 5 South Asian countries in the period 1990–2014. They witnessed the positive impacts of renewable and nonrenewable energy utilization and fixed capital formation on economic growth. Besides, the study also supported the conservation hypothesis in South Asian countries.

## 3 Empirical model, data, and methodology

### 3.1 Empirical model

The main objective of this research is examining the roles of renewable and nonrenewable power usage consumption as one of the determinants of sustainable development in African EMDEs. The empirical model used in this study is based on the baseline model of the Cobb-Douglas production function, as follows:

(1) $$Y=AK^{\alpha_{1}}L^{\alpha_{2}}$$

where Y is output, A is technological factor, K is capital, and L is labour. $\alpha_{1}$ and $\alpha_{2}$ respectively denote the elasticities of output to capital and labour.

According Shahbaz et al. (2013), Kumar et al. (2014), Kahia et al. (2017) and Le and Sarkodie, 2020, in the extended Cobb-Douglas production function, technology can be endogenously determined by energy use, trade openness, and government expenditures. Therefore, Eq. (1) can be defined as follows:

(2) $$Y=K^{\alpha_{1}}L^{\alpha_{2}}NRE^{\alpha_{3}}RE^{\alpha_{4}}GC^{\alpha_{5}}TO^{\alpha_{6}}$$

where NRE and RE stand for nonrenewable and renewable energy use, respectively; GC and TO represent government expenditure and trade openness, respectively.

Transform Eq. (2) in per capita terms and turning it into the log-linear specification, the empirical model for panel data sample as follows:

(3) $$lnGDP_{it}=\alpha_{it}+\beta_{1}lnGCF_{it}+\beta_{2}lnNRE_{it}+\beta_{3}lnRE_{it}+\beta_{4}lnGC_{it}+\beta_{5}lnTO_{it}+\varepsilon_{it}$$

where GDP is GDP per capita, α is constant, $\beta_{1},\beta_{2},\beta_{3},\beta_{4}\mathrm{and}\beta_{5}$ respectively denote the elasticity coefficients of gross fixed capital formation per capita (lnGCF), non-renewable power consumption per capita (lnNRE), renewable power consumption per capita (lnRE), general government final consumption expenditure per capita (lnGC) and trade openness per capita (lnTO). The notations *i* and *t* indicate country and year respectively. The final element, $\varepsilon_{\mathrm{it}}$, is the error term.

### 3.2 Data and descriptive statistics

This work uses balanced panel data with 500 observations between the years 1990 and 2014 of 20 African Emerging Market and Developing Economies classified by Morgan Stanley Capital Income including Algeria, Arab Republic of Egypt, Islamic Republic of Iran, Jordan, Lebanon, Morocco, Saudi Arabia, Tunisia, Botswana, Cameroon, Republic of the Congo, Cote d'Ivoire, Gabon, Ghana, Kenya, Mozambique, Nigeria, Senegal, Sudan and Tanzania.

The data is collected from two sources, subject to the availability of the data. First, GDP, capital, trade openness, and government expenditure are provided by World Development Indicators (WDI) database of the World Bank (2018). Second, conventional fuels consumption (billions of kilowatt-hours) and renewable power usage (billions of kilowatt-hours) are downloaded from Energy Information Administration (EIA). All variables are computed under per capita and then transformed into natural logarithm. Table 2 provides detailed description of the selected variables along with their sources.

**Table 2:** The symbol of variables and sources.

Symbol	Explanation	Source
lnGDP	Per capita GDP, in term of the natural logarithm.	WDI
lnGCF	Per capita capital, in term of the natural logarithm.	WDI
lnNRE	Per capita conventional fuels consumption from oil, coal, and gas, in term of the natural logarithm.	EIA
lnRE	Per capita renewable power consumption from hydropower, wind power, solar, modern biofuels, geothermal, wave and tidal, in term of the natural logarithm.	EIA
lnGC	Per capita government expenditure, in term of the natural logarithm.	WDI
lnTO	Per capita trade openness, in term of the natural logarithm.	WDI

Table 3 provides the descriptive statistical analysis of all variables.

**Table 3:** Descriptive statistics.

Variables	Mean	St.D	Min	Max
lnGDP	7.7020	0.9968	5.0866	9.9610
lnGCF	6.1931	1.1508	2.9952	8.5820
lnNRE	0.8053	1.3122	-1.7029	3.7514
lnRE	-0.0982	1.9659	-6.0961	3.3935
lnGC	5.7146	1.2156	2.5244	8.8313
lnTO	7.2605	1.2107	4.3373	9.8533

The correlation matrix of the variables is given in Table 4.

**Table 4:** Correlation matrix and variance inflation factor (VIF) statistics.

	lnGDP	lnGCF	lnNRE	lnRE	lnGC	lnTO
lnGDP	1					
lnGCF	0.7700	1				
lnNRE	0.8708	0.4270	1			
lnRE	0.4529	0.5898	-0.7609	1		
lnGC	0.8385	0.3992	0.2171	-0.2426	1	
lnTO	0.7622	0.2448	0.3387	-0.1458	0.3416	1
Tolerance		1.961	2.781	1.717	1.125	1.876
VIF		0.510	0,360	0.582	0.889	0.533

The correlation matrix of the variables demonstrates the positive correlation among renewable and nonrenewable energy usage, government expenditure and trade openness. We also examine the level of multicollinearity among the variables, and the VIF coefficients show that multicollinearity is not a problem in our estimation.

### 3.3 Econometric methodology

Traditional econometric techniques ignore the existence of CSD and slope heterogeneity. Thus, when CSD and slope heterogeneity occur, first-generation econometric methods can be biased and unreliable (Grossman and Krueger, 1995; Pesaran, 2004). To avoid such problem, after the presence of CSD and slope heterogeneity is confirmed, this study employs second-generation econometric techniques for subsequent analyses. The estimation procedure consists of 6 steps, which is summarized as follows:

*First*, the author conducts CSD test and the slope homogeneity test respectively proposed by Pesaran (2004) and Pesaran and Yamagata (2008).

*Second*, in case CSD appears, the cross-sectionally augmented Dickey-Fuller (CADF) and the cross-sectionally augmented Im-Pesaran-Shin (CIPS) panel unit root test will be utilized to check for the stationary properties of variables. The aforementioned unit root tests are deemed second-generation ones which were developed by Pesaran (2007).

*Third*, to check the cointegrated relationship among the variables, the author employs Westerlund (2007) cointegration test.

*Fourth*, after affirming the cointegration of the variables, the author applies the panel AMG estimator (Eberhardt and Bond, 2009; Eberhardt and Teal, 2010) to scrutinize the long-run impacts of nonrenewable and renewable energy use, government expenditure, capital and trade openness on economic growth in African EMDEs.

*Fifth*, for the purpose of robustness check, the authors will use the MG estimator (Pesaran and Smith, 1995) and the CCEMG estimator of Pesaran (2006).

*Finally*, the author will analyze the causality among variables using Dumitrescu and Hurlin's Granger tests (Dumitrescu and Hurlin, 2012).

#### 3.3.1 Cross-sectional dependence test

Checking CSD in panel data is very important because it determines whether first-generation or second-generation econometric techniques are applied. While the first-generation econometric techniques may give misleading results due to ignoring CSD, second-generation ones are considered appropriate when accounting for CSD (Pesaran, 2004).

To test cross-sectional dependence, Pesaran (2004) relied on the following statistic:

(4) $$CSD=\sqrt{\frac{2T}{N(N-1)}}\sum_{i=1}^{N-1}\sum_{j=i+1}^{N}{\hat{\rho }}_{ij}$$

where ${\hat{\rho }}_{\mathrm{ij}}$ is a correlation between the errors.

The result of the test, indicated by the comparison between p-value and significance level, helps determine whether CSD occurs in the panel data. Particularly, when p-value is smaller than significance level, there is evidence for the existence of CSD, and thus the null hypothesis (i.e. no cross-sectional dependence) is rejected. Otherwise, we do not reject the null hypothesis.

#### 3.3.2 Slope homogeneity test

In panel data analysis, it is necessary to inspect if cross-sectional heterogeneity occurs because when the panel is heterogeneous, the assumption of slope homogeneity might create misleading estimation (Breitung, 2005). To test for slope homogeneity, we employ the work of Pesaran and Yamagata (2008) with the following test statistics:

(5) $$S=\sum_{i=1}^{N}{\left(\beta_{i}-\beta_{WFE}\right)}^{'}\frac{X_{i}^{'}M_{\tau }X_{i}}{\sigma_{i}^{2}}\left(\beta_{i}-\beta_{WFE}\right)$$

(6) $$\bar{\Delta }=\sqrt{N}\left(\frac{N^{-1}S-k}{\sqrt{2k}}\right)$$

(7) $${\bar{\Delta }}_{adj}=\sqrt{N}\left(\frac{N^{-1}S-k}{\sqrt{\frac{2k\left(T-k-1\right)}{T+1}}}\right)$$

where S and $\bar{\Delta }$ are the test statistics; ${\bar{\Delta }}_{adj}$ is the biased-adjusted version of the $\bar{\Delta }$ test; $\beta_{i}$ denotes the pooled OLS coefficient; $\beta_{WFE}$ indicates the weighted fixed effect (WFE) pooled estimator; $X_{i}$ is the matrix containing explanatory variables in deviations from the mean; $M_{\tau }$ is the identity matrix; $\sigma_{i}^{2}$ is the estimate of $\sigma_{i}$, and k indicates the number of regressors.

#### 3.3.3 Panel unit root test

To test for the stationarity of the variables in the panel data with cross-sectional dependence, Pesaran (2007) developed the cross-sectionally augmented Dickey-Fuller (CADF) and the cross-sectional augmented IPS (CIPS) tests. The following regression represents the CADF:

(8) $$\Delta y_{i,t}=\pi_{i}+\rho_{i}y_{i,t-1}+\sigma_{i}\Delta {\bar{y}}_{i,t}+\tau_{i}{\bar{y}}_{t-1}+\mu_{i,t}$$

where $\Delta {\bar{y}}_{i,t}=\frac{1}{N}\sum_{i=1}^{N}\Delta y_{i,t};{\bar{y}}_{t-1}=\frac{1}{N}\sum_{i=1}^{N}y_{i,t-1}$ and $\mu_{i,t}$ is the error term.

The CIPS test is stated as:

(9) $$CIPS=\frac{1}{N}\sum_{i=1}^{N}CADF_{i}$$

where $CADF_{i}$ indicates the CADF statistic in Eq. (8).

The results of CADF test and CIPS test will indicate the stationarity of the variables. Specifically, when the test statistics are bigger than the critical values, we reject the null hypothesis and conclude the stationary properties of the variables. Otherwise, we cannot reject the null hypothesis (i.e. the data has unit root).

#### 3.3.4 Panel cointegration test

Common techniques, such as Pedroni (1999) and Kao (1999), are often used for testing cointegration in panel data. However, in the presence of cross-sectional dependence, the aforesaid first-generation cointegration tests might generate biased results because they rely on cross-sectional-independence assumption (Westerlund, 2007). Accounting for the CSD, Westerlund (2007) proposed the cointegration test based on the error correction model as follows:

(10) $$\Delta y_{i,t}=\sigma_{i}^{'}d_{t}+\theta_{i}\left(y_{i,t-1}-\beta_{i}^{'}x_{i,t-1}\right)+\sum_{j=1}^{k}\phi_{ij}\Delta y_{i,t-j}+\sum_{j=0}^{k}\delta_{ij}\Delta x_{i,t-j}+\varepsilon_{i,t}$$

where $\theta_{i}=$ the error correction term for ith individual.

The Westerlund (2007) test's null hypothesis assumes zero error correction term (in a conditional error correction specification of the panel data) and indicates no cointegration among the variables.

Westerlund (2007) provided 4 statistics including $G_{\tau },G_{\alpha },\;P_{\tau }$, and $P_{\alpha }$ statistics. The $G_{\tau }$ and $G_{\alpha }\;$statistics help detect cointegration in one or more cross-sectional units, while the $P_{\tau }$ and $P_{\alpha }\;$statistics help detect cointegration in the whole panel. Their formulas are given as:

(11) $$G_{\tau }=\frac{1}{N}\sum_{i=1}^{N}\frac{{\hat{\theta }}_{i}}{SE\left({\hat{\theta }}_{i}\right)}\;$$

(12) $$G_{\alpha }=\frac{1}{N}\sum_{i=1}^{N}\frac{T{\hat{\theta }}_{i}}{1-\sum_{j=1}^{k}{\hat{\theta }}_{ij}}\;$$

(13) $$P_{\tau }=\frac{\hat{\theta }}{SE\left(\hat{\theta }\right)}\;$$

(14) $$P_{\alpha }=T\hat{\theta }$$

#### 3.3.5 Heterogeneous parameter estimates

To estimate the long-run parameters in heterogeneous panel data, Eberhardt và Bond (2009) introduced the Augmented Mean Group (AMG) estimator, which allows both cross-sectional dependence and slope heterogeneity. The AMG estimator is deemed a highly robust calculation tool. It is computed through 2 steps.

The first step is combining the unobserved common factor with the time dummies in the following equation:

(15) $$\Delta y_{it}=\pi_{i}+\rho_{i}\Delta x_{it}+\sigma_{i}f_{t}+\sum_{t=1}^{T}\theta_{t}D_{t}+\varepsilon_{it}$$

where Δ represents the first difference operator, $\pi_{i}$ indicates the intercept; $y_{\mathrm{it}}$ and $x_{\mathrm{it}}$ are dependent and independent variables respectively; $\rho_{i}$ denotes the slope of each unit; $f_{t}$ denotes the unobserved common factor; $\sigma_{i}$ is the heterogeneous factor loadings; D and θ are the time dummies and their coefficients respectively; and $\varepsilon_{\mathrm{it}}$ is the error term.

The second step is obtaining the MG estimator for AMG by averaging the slopes of each unit:

(16) $$AMG=\frac{1}{N}\sum_{i=1}^{N}{\hat{\rho }}_{i}$$

where ${\hat{\rho }}_{i}$ are the estimates of $\rho_{i}$ in Eq. (15).

It is noticeable that the AMG estimator remains efficient and unbiased regardless of the cross-section and time dimensions in panel data (Bond and Eberhardt, 2013). For robustness check, this study will also use the MG estimator (Pesaran and Smith, 1995) and the CCEMG estimator of Pesaran (2006).

#### 3.3.6 Panel causality tests

To test causality in heterogeneous panel data, Dumitrescu and Hurlin (2012) proposed the linear model described as follows:

(17) $$y_{i,t}=\alpha_{i}+\sum_{k=1}^{K}\delta_{ik}y_{i,t-k}+\sum_{k=1}^{K}\theta_{ik}x_{i,t-k}+\varepsilon_{i,t}$$

where $\alpha_{i}$ is individual fixed effects, $\delta_{\mathrm{ik}}$ and $\theta_{\mathrm{ik}}\;$represent the lag parameters and slope parameters, respectively, K is lag length.

The null hypothesis of Dumitrescu and Hurlin causality test is no causation in the panel. The alternative hypothesis states that there exists causal relationship in at least a unit. The hypotheses are as follows:

(18) $$H_{0}:\theta_{i1}=\ldots =\theta_{iK}=0,\forall i=1,\ldots ,N$$

(19) $$H_{1}:\theta_{i1}=\ldots =\theta_{iK}=0,\forall i=1,\ldots ,N_{1}$$

(20) $$\theta_{i1}\neq 0or\ldots or\theta_{iK}\neq 0,\forall i=N_{1}+1,\ldots ,N$$

The result of Dumitrescu and Hurlin causality test is indicated by the comparison between p-values and significance levels. If the former are smaller than the latter, the null hypothesis is rejected. In contrast, the null hypothesis cannot be rejected.

Dumitrescu and Hurlin proposed the Wald test statistic ($\bar{W})$ as described in the following equation:

(21) $$\bar{W}=\frac{1}{N}\sum_{i=1}^{N}W_{i}$$

in which $W_{i}$ denotes the individual Wald statistics for each cross-section unit at time T.

## 4 Results

### 4.1 Results of cross-sectional tests

In the first step of panel data analysis, we check if CSD exists in the data. This step is very vital because it decides whether first- or second-generation econometric methods will be used in subsequent analyses. The results of CSD test proposed by Pesaran (2004) are demonstrated in Table 5.

**Table 5:** Results of CSD test.

Variable	CD-test	P-value	Corr	Abs(corr)
lnGDP	35.131∗∗∗	0.000	0.51	0.75
lnGCF	20.773∗∗∗	0.000	0.30	0.45
lnNRE	30.920∗∗∗	0.000	0.45	0.51
lnRE	16.210∗∗∗	0.000	0.29	0.47
lnGC	12.689∗∗∗	0.000	0.18	0.45
lnTO	34.297∗∗∗	0.000	0.50	0.61

Notes: The symbol ∗∗∗ indicates that p-value is smaller than 1%.

The results in Table 5 indicate that the null hypothesis is rejected for all variables at 1% level. It is evidenced that CSD exists in GDP per capita, gross fixed capital formation per capita, non-renewable power consumption per capita, renewable power consumption per capita, general government final consumption expenditure per capita, and trade openness per capita. Thus, second-generation econometric techniques should be utilized for further estimation.

### 4.2 Results of slope homogeneity test

The test of Pesaran and Yamagata (2008) is employed to examine the presence of slope heterogeneity in the data. The outcomes in Table 6 indicate that the null hypothesis (slope homogeneity) is rejected for all variables. Accordingly, the data has slope heterogeneity issue.

**Table 6:** The results of slope homogeneity test.

Variable	$\bar{\Delta }$	${\bar{\Delta }}_{adj}$
lnGDP	75.702∗∗∗	98.56∗∗∗
lnGCF	49.788∗∗∗	106.01∗∗∗
lnNRE	52.328∗∗∗	103.26∗∗∗
lnRE	265.575∗∗∗	288.34∗∗∗
lnGC	107.073∗∗∗	216.66∗∗∗
lnTO	84.972∗∗∗	138.66∗∗∗

Notes: ∗∗∗ indicates that p-value is smaller than 1%.

### 4.3 Results of panel unit roots

As CSD and slope heterogeneity are detected, second-generation unit root test should be used. This study applies the CADF and CIPS tests of Pesaran (2007) to check the stationarity of the variables. The results in Table 7 show that all variables are stationary at first difference.

**Table 7:** Results from stationary properties in the panel.

Variables	CADF		CIPS	
	Level	Δ	Level	Δ
lnGDP	–1.895	–2.786∗∗∗	–1.739	–4.041∗∗∗
lnGCF	–1.802	–3.463∗∗∗	–1.864	–4.473∗∗∗
lnNRE	–1.855	–3.193∗∗∗	–2.109	–5.261∗∗∗
lnRE	–1.119	–3.551∗∗∗	–1.488	–4.917∗∗∗
lnGC	–1.921	–3.333∗∗∗	–2.036	–4.605∗∗∗
lnTO	–1.972	–3.296∗∗∗	–1.955	–4.620∗∗∗

Notes: The symbol ∗∗∗ indicates that p-value is smaller than 0.01.

### 4.4 Results of panel cointegration tests

Westerlund (2007) cointegration test is now conducted. The results in Table 8 indicate that the null hypothesis (no cointegration) is rejected, which confirms the long-run relationship among nonrenewable and renewable energy usage, government expenditure, gross fixed capital formation, trade openness and economic growth in African EMDEs.

**Table 8:** The results of cointegration test.

Stat.	Value	Z-value	Robust P-value
$G_{\tau }$	–2.939∗∗∗	–2.469	0.007
$G_{\alpha }$	–9.822∗∗	3.144	0.000
$P_{\tau }$	–18.627∗∗∗	–7.213	0.000
$P_{\alpha }$	–12.561∗∗∗	–3.651	0.000

Notes: The symbol ∗∗ and ∗∗∗ respectively indicate that p-value is smaller than 5% and 1% levels.

### 4.5 The long-run estimation results

After the cointegration of the variables is verified, the author employs the panel AMG estimator (Eberhardt and Bond, 2009; Eberhardt and Teal, 2010), the MG estimator (Pesaran and Smith, 1995) and the CCEMG estimator (Pesaran, 2006) to estimate long-run coefficients. Detailed results are displayed in Table 9.

**Table 9:** Long-run estimation.

Regressors	AMG estimator		MG estimator		CCEMG estimator	
	Coefficient	t-stat.	Coefficient	t-stat.	Coefficient	t-stat.
lnGCF	0.151∗∗∗	4.93	0.153∗∗∗	4.26	0.146∗∗∗	3.12
lnNRE	0.129∗∗∗	4.39	0.130∗∗∗	5.21	0.124∗∗∗	2.73
lnRE	0.048∗∗∗	4.56	0.040∗∗∗	2.82	0.052∗∗∗	3.26
lnGC	0.125∗∗∗	3.15	0.120∗∗∗	3.46	0.128∗∗∗	4.68
lnTO	0.067∗∗	2.29	0.066∗∗	2.45	0.058∗∗	2.06

Notes: The symbol ∗∗, ∗∗∗ indicate significance at the 5% and 1%.

The estimation results demonstrate that, in the long-run, gross fixed capital formation per capita and the government expenditure per capita stimulate GDP per capita by 0.151% and 0.125% respectively per 1% increase. These findings point out that capital formation has always been a crucial input of an economy's production, and thus, more investment in infrastructure helps boost production capacity and create positive effects for economic growth. Meanwhile, government expenditure seems to be an important factor in economic growth. Government intervention is needed in order to successfully converting nonrenewable energy sources into renewable ones. Government expenditure can bring innovation in production processes by reducing energy usage, which in turn lowers carbon amount. Moreover, government expenditure on investment subsidies, credit incentives, tax incentives, standard and quota establishment, and green transfers can facilitate renewable energy development.

Besides, the trade-led growth effect in African EMDEs is also evidenced when GDP per capita rises 0.067% due to 1% improvement of trade openness. The positive impacts of trade openness on economic growth are contributed by the increase in the trade of goods and services as well as the transfer of advanced technology from developed economies to African emerging countries. The governments of African EMDEs need to build green trade policies and promote trade activities providing technology-transfer channels from developed countries in order to reduce production cost as well as stimulate production capacity from renewable sources. Besides, in pursuing sustainable development objective, policy-makers in African EMDEs must limit the import of fossil fuels because it can cause negative influences on environmental quality and trade balance.

The main focused result of this study is the impact of energy-consumption structure and economic growth. We observe that renewable and nonrenewable energy sources positively affect economic growth in the long-run at 1% significance level, which implies that more power usage will encourage economic growth. Specifically, 1% increase of nonrenewable energy usage per capita boosts GDP per capita by 0.129%. Meanwhile, GDP per capita rises by 0.048% per 1% increase of renewable energy consumption per capita. This supports the *(i)* research hypothesis regarding the positive impacts of renewable and nonrenewable energy usage on economic growth. Although the findings show that the contribution of renewable energy consumption to economic growth is still not as big as the nonrenewable one, our study supports the economic benefits of activating relevant environmentally-friendly energy sources. In the long-run, renewable energy can lead to economic sustainability when the nonrenewable sources are limited over time. Moreover, regarding the sustainable development objective, it is possible to find clean, reasonably-cheap, available and easily-accessible-to-everybody energy from renewable sources because their distribution is dispersed and they can solve the energy imbalance between rural and urban areas (Brew-Hammond, 2010). In addition, renewable energy brings other benefits which reduce energy import, contribute to the diversification of energy supply, minimize the risk of price fluctuation and create opportunities for improving power security (Owusu and Asumadu, 2016). Besides, in comparison with the fossil fuels, renewable energy diminishes carbon dioxide emissions, thus protecting the environment and health (Asumadu-Sarkodie and Owusu, 2016).

The signs and magnitudes of the coefficients computed by AMG, MG and CCEMG estimators are consistent and very similar, which signifies the robustness of estimation.

### 4.6 Results of panel causality tests

In order to effectively assess the causation among the variables in the presence of cross-sectional dependence and slope heterogeneity, the author uses Dumitrescu and Hurlin (2012) Granger causality test. The outcomes are demonstrated in Table 10.

**Table 10:** Outcome of Granger causality tests.

Variables	lnGDP	lnGCF	lnNRE	lnRE	lnGC	lnTO
lnGDP	–	8.6578∗∗∗	5.1351∗∗∗	6.6735∗∗	8.4662∗∗∗	7.3176∗∗∗
		(19.9222)	(5.0572)	(2.3667)	(3.6516)	(3.2776)
lnGCF	6.5856∗∗∗	–	6.6369∗∗∗	2.8767∗∗	4.1418∗∗∗	7.3794∗∗∗
	(7.5999)		(7.6898)	(4.6726)	(8.0099)	(5.1402)
lnNRE	5.6861∗∗∗	4.6057∗∗∗	–	1.7940∗∗	2.5076∗∗∗	5.8622∗∗∗
	(6.0231)	(4.1294)		(2.5109)	(3.6992)	(6.3319)
lnRE	4.2305∗∗∗	2.2162∗∗∗	9.6681∗∗∗	–	2.2698∗∗∗	2.9466∗∗∗
	(8.2439)	(2.9303)	(8.1429)		(3.0718)	(4.8571)
lnGC	5.0051∗∗∗	2.7973∗∗∗	4.7055∗∗∗	2.5081∗∗∗	–	3.5923∗∗∗
	(10.2871)	(4.4632)	(4.3042)	(3.7005)		(6.5604)
lnTO	2.6193∗∗∗	2.3147∗∗∗	1.9822∗∗	2.2328∗∗∗	3.2771∗∗∗	–
	(3.9938)	(3.1904)	(2.3132)	(2.9742)	(5.7290)	

Notes: The W-statistics marked with ∗∗∗ are significant at 1% level. Z-statistics are shown in parentheses.

According to the results in Table 10, it can be witnessed that there exist two-way relationship between nonrenewable energy usage and economic growth as well as between renewable energy usage and economic growth, thus demonstrating the presence of the feedback hypothesis between the two components of energy consumption (nonrenewable fuels and renewable power) and economic growth. Consequently, the *(ii)* research hypothesis is validated. Based on the feedback hypothesis, it is implied that the increase in power consumption can facilitate economic growth, and vice versa, economic growth can boost energy usage, which signifies that energy conservation policies can lower economic growth efficiency; therefore, the transition in the energy consumption structure from nonrenewable to renewable sources is a useful solution for African EMDEs' sustainable development. African EMDEs can raise the penetration of renewable energy sources in their power system in the situation that fossil-fuel utilization stimulates carbon dioxide emissions and other problems relating to severe climate change. Besides, the findings also demonstrate the feedback mechanism between renewable energy consumption and nonrenewable energy consumption, which implies that a strategic combination of producing and consuming these two kinds of power in an energy portfolio can help achieve reasonably-priced and widely-accessible energy services as well as the reduction in climate change and its consequences.

## 5 Conclusion

African EMDEs have integrated quickly to the global economy; however, they face the challenges of energy security and environmental degradation. This study aims to find the ways in which African EMDEs can pursue green economy through constructing effective power policies in the circumstance of sustainable development goal. So as to attain the research objective, we build the model to assess the long-term impacts of energy components, along with other factors, on economic growth. In addition, the causation between energy components in energy consumption structure and economic growth is evaluated by Dumitrescu and Hurlin causality analysis. Empirically, this study employs the extended production function considering the roles of nonrenewable fuels usage and renewable power consumption alongside important factors such as capital, trade openness, and government expenditure in the economic growth. The data is balanced and covers the period 1990–2014 for African EMDEs. Due to the presence of slope heterogeneity and cross-sectional dependence, this work utilizes second-generation econometric techniques for heterogeneous panel. Accordingly, CADF and CIPS tests are used for checking the stationarity of the variables, and the outcomes indicate that all variables are stationary at first difference. Next, Westerlund panel cointegration test is conducted, and the author detects the cointegration between economic growth and other independent variables. The long-run estimation based on Augmented Mean Group (AMG) estimator demonstrates that nonrenewable energy use, renewable energy consumption, capital, trade openness, and government expenditure positively impact economic growth in African EMDEs. The robustness of estimation is confirmed by CCEMG and MG estimators. Moreover, this study validates the feedback hypothesis (two-way causality) between each component of energy usage and economic growth in African EMDEs.

We observe that both renewable and nonrenewable energy consumption positively affect economic growth in the long-run. Our results are consistent with prior studies such as Apergis and Payne (2011) for 80 countries, Tugcu et al. (2012) for G7, Al-mulali (2014) for 18 Latin American countries, Jebli and Youssef (2015) for 69 countries, Kahia et al. (2017) for MENA Net Oil Importing Countries, Gozgor et al. (2018) for 29 OECD Countries, Le and Sarkodie, 2020 for 45 EMDEs, Le and Bao (2020) for 16 Latin American and Caribbean countries, and Rahman and Velayutham (2020) for 5 South Asian countries. Meanwhile, Ahmed et al. (2019) reported that renewable energy fostered economic growth in Myanmar while the nonrenewable power had opposite effect. Further, the findings also indicate bidirectional connection between renewable power usage and economic growth as well as between nonrenewable energy utilization and economic growth. In other words, our study acknowledges the presence of the feedback hypothesis in case of the African EMDEs, which is in line with Apergis and Payne (2011), Al-mulali (2014), Kahia et al. (2017), Bekun et al. (2019), Le and Sarkodie, 2020, Le and Bao (2020). Our results have some differences with some researches. Namely, Payne (2009) and Jebli and Youssef (2015) reported that there was no causation between energy components and economic growth (i.e. supported neutrality hypothesis). Ahmed et al. (2019) documented the growth hypothesis for the association between both types of energy usage and economic growth in Myanmar. Ben Mbarek et al. (2018) concluded that the growth hypothesis applies for the linkage between renewable energy utilization and economic growth while the conservation hypothesis occurs in the connection between nonrenewable energy usage and economic growth in Tunisia. Rahman and Velayutham (2020) found that the conservation hypothesis is valid for South Asian countries.

From our findings, it can be observed that both renewable and nonrenewable energy are important to economic growth whey they foster economic growth in the long-run. Hence, energy conservation policies can lower economic growth efficiency. Our results imply that policy-makers should focus not only on the increase of renewable energy but also the increase of nonrenewable power in order to support economic growth. Besides, the interdependence of renewable energy, nonrenewable power and economic growth demonstrates that energy is crucial for economic growth, and economic growth also boosts energy consumption. It is important to notice that utilizing different energy sources can facilitate economic growth and reduce environmental degradation when renewable energy is beneficial in decelerating climate change and suiting sustainable development objectives (Asumadu-Sarkodie and Owusu, 2016). Consequently, sustainable energy policies should foster the transition in the structure of energy production and consumption by reducing the proportion of nonrenewable sources and increasing the share of renewable ones without harming the growth pattern. Furthermore, the selected African EMDEs should modernize the production in order to use energy efficiently and minimize carbon dioxide emissions. This necessitates disruptive technological innovation in order to reduce energy usage (both renewable and nonrenewable) and simultaneously increase the utilization efficiency. More specifically, African EMDEs need more cooperation, research and development as well as innovation towards expanding infrastructure, upgrading and developing sustainable power technology and supplementary technology, which minimizes climate change and the relating consequences. Besides, policy-makers should introduce and provide effective mechanism to motivate the development of renewable energy projects and encourage the private sector to participate in implementing these projects or promote public-private partnership. Additionally, along with trade policies and green technology transfer, the governments of African EMDEs need to establish legal frameworks for power security and strong environmental protection through government expenditure. Policy-makers of African EMDEs should effectively conduct the aforementioned policies combined with socio-economic policies that treat power security as one of the most important focuses of sustainable development.

In this study, we use non-renewable and the renewable energy consumption at the aggregated level for discovering the power consumption – economic growth linkage. Hence, we suggest that further researches in the energy-growth nexus can focus on the renewable energy consumption at the disaggregated level (e.g. the geothermal, the biomass, the solar, the tides, the wind, and biofuels) and the nonrenewable energy consumption (e.g. natural gas, oil, coal, and nuclear). Besides, future researches can implement detailed examination of the relationship between energy usage and economic growth by disaggregating data into different industries in order to clearly assess the energy policies that contribute to the sustainable development objective in African EMDEs. Moreover, researches conducted at country level are also helpful for designing specific sustainable development for each country in African EMDEs. Finally, in the future, subject to the availability and reliability of data, it can be very interesting that studies with the same topic can consider different control variables as well as more advanced models and econometric techniques.

## Declarations

### Author contribution statement

Hoang Phong Le & Dang Thi Bach Van: Conceived and designed the experiments; Performed the experiments; Analyzed and interpreted the data; Contributed reagents, materials, analysis tools or data; Wrote the paper.

### Funding statement

This work was supported by the European Union's Horizon 2020 research and innovation programme under Marie Skłodowska-Curie grant agreement No. 734712. This work was also supported by the 10.13039/100007839University of Economics Ho Chi Minh City.

### Competing interest statement

The authors declare no conflict of interest.

### Additional information

No additional information is available for this paper.
